# Regulation of Microtubule Dynamics in Axon Regeneration: Insights from *C. elegans*

**DOI:** 10.12688/f1000research.8197.1

**Published:** 2016-04-27

**Authors:** Ngang Heok Tang, Andrew D. Chisholm

**Affiliations:** 1 Section of Neurobiology, Division of Biological Sciences, University of California, San Diego, La Jolla, CA, 92093, USA

**Keywords:** DLK-1, EFA-6, PAR-1/MARK

## Abstract

The capacity of an axon to regenerate is regulated by its external environment and by cell-intrinsic factors. Studies in a variety of organisms suggest that alterations in axonal microtubule (MT) dynamics have potent effects on axon regeneration. We review recent findings on the regulation of MT dynamics during axon regeneration, focusing on the nematode Caenorhabditis elegans. In *C. elegans* the dual leucine zipper kinase (DLK) promotes axon regeneration, whereas the exchange factor for Arf6 (EFA-6) inhibits axon regeneration. Both DLK and EFA-6 respond to injury and control axon regeneration in part via MT dynamics. How the DLK and EFA-6 pathways are related is a topic of active investigation, as is the mechanism by which EFA-6 responds to axonal injury. We evaluate potential candidates, such as the MT affinity-regulating kinase PAR-1/MARK, in regulation of EFA-6 and axonal MT dynamics in regeneration.

## Introduction

More than a hundred years ago, Ramon y Cajal was the first to describe how individual axons respond to injury ^1^. Many types of axons regenerate, including neurons in the peripheral nervous system (PNS), re-forming growth cones similar to those that Ramon y Cajal had characterized during development. In contrast, neurons of the mammalian central nervous system (CNS) often fail to regenerate, and their damaged ends form swollen, non-motile structures later termed retraction bulbs. These fundamental observations set the stage for subsequent exploration of why regenerative capacity varies drastically between the CNS and PNS. Many studies have focused on the inhibitory environment of the adult mammalian CNS ^2– 4^. However, it is becoming evident that cell-intrinsic processes are also key determinants of axon regeneration ^5^. Among these intrinsic factors, regulation of axonal microtubule (MT) dynamics has emerged as a major influence on the capacity of an axon to regrow effectively ^6– 10^. Most strikingly, pharmacological stabilization of MTs by paclitaxel or related molecules enhances axon regeneration *in vitro* and *in vivo*, suggesting a potentially therapeutically significant role for MT dynamics in axon regeneration ^11– 13^.

**Figure 1.**
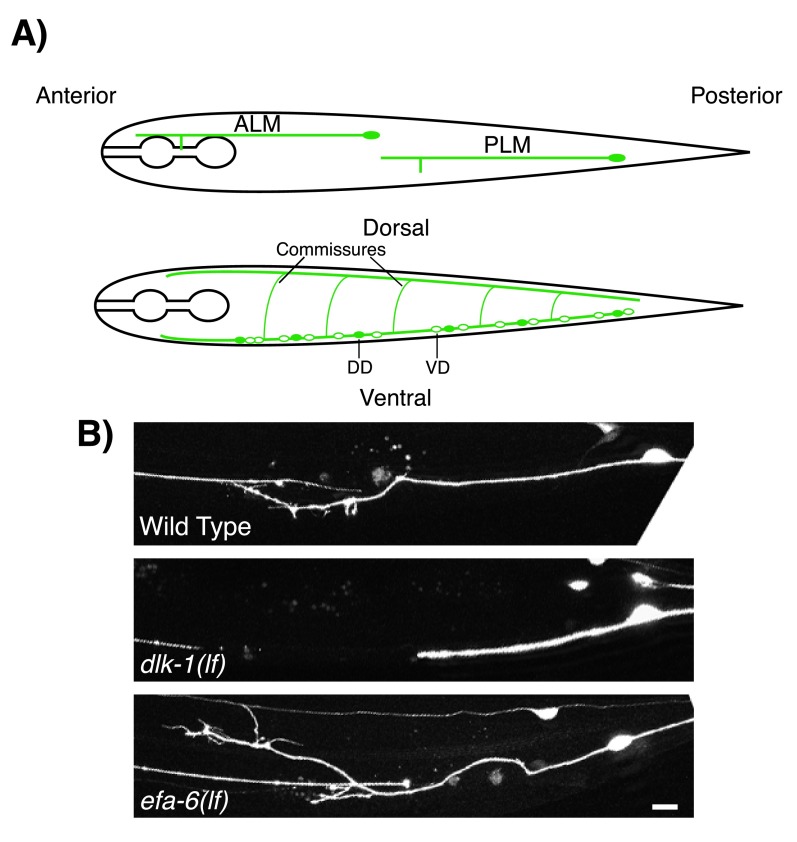
DLK-1 and EFA-6 regulate axon regeneration after injury in *Caenorhabditis elegans*. ( **A**) Illustration of the positions of the mechanosensory neurons anterior lateral microtubule (ALM) and posterior lateral microtubule (PLM) cells (top) and the GABAergic motor neurons dorsal D (DD) and ventral D (VD) (bottom). ( **B**) Examples of PLM axon regeneration in wild-type, *dlk-1(lf)*, and *efa-6(lf)* at 24 hours post-axotomy. *dlk-1(lf)* mutant (middle) shows decreased axon regeneration, whereas *efa-6(lf)* mutant (bottom) shows increased axon regeneration upon axotomy, compared with wild-type (top). Scale bar, 20 µm. DLK, dual leucine zipper kinase.

The nematode *C. elegans* has long been used for studies in neuronal development and behavior, owing to its short life cycle, genetic tractability, and ease of *in vivo* imaging. The nervous system of an adult *C. elegans* hermaphrodite consists of 302 neurons of nearly invariant lineage ^14^. A decade ago, a pioneering study showed that axons of mature *C. elegans* neurons can regenerate after precise laser axotomy ^15^. Several kinds of neuron display robust axon regeneration; most work has focused on the mechanosensory and motor axons ( Figure 1A) ^16, 17^. The genetic tractability and ease of imaging *in vivo* have made *C. elegans* a rising star in axon regeneration studies. Several laboratories have used large-scale genetic ^18^, chemical ^19^, and RNA interference (RNAi) ^20^ screens to identify genes or molecules that regulate axon regeneration. These studies have identified several new players in axon regeneration, including the dual leucine zipper kinase (DLK) and mixed lineage kinase (MLK) mitogen-activated protein kinase (MAPK) pathways ^21, 22^, Notch signaling pathway ^23^, insulin signaling pathway ^24^, and microRNA ^25^. Some pathways, such as DLK signaling, have been shown to function in axon regeneration in vertebrates ^26– 29^, suggesting that axon regeneration factors identified in *C. elegans* may be suitable for translational studies. Here, we review the role of MT dynamics in axon regeneration, primarily focusing on *C. elegans*.

## Axonal microtubule organization before and after injury

MTs are among the major cytoskeletal structures in cells. MTs are cylindrical and polarized polymers formed by αβ-tubulin heterodimers arranged in a head-to-tail configuration ^30^. *In vitro* and *in vivo* MTs undergo rapid growth (i.e. polymerization) and shrinkage (i.e. depolymerization) at their plus ends, a behavior known as dynamic instability ^31^. Minus ends of MTs are relatively stable but can also undergo polymerization and depolymerization. MT dynamics *in vivo* are influenced by many factors, including concentration of free tubulin monomers and tubulin post-translational modifications, and by MT-binding proteins. MT plus-end dynamics are regulated by a large cohort of plus end-tracking proteins (+TIPs) ^32^; relatively few minus end-targeting proteins (−TIPs) have been identified that regulate MT minus ends ^33^. Together, these proteins affect the frequency of catastrophe (switching from growth to shrinkage) and rescue (switching from shrinkage to growth) events.

In contrast to the highly dynamic behavior of MTs in dividing or migrating cells, axonal MTs of mature neurons are relatively stable, forming a consistent architecture that maintains neuronal polarity and allows directed axonal transport ^34^. Axonal MTs in *C. elegans* were first characterized in the MT-rich mechanosensory neurons ^35, 36^. More recent imaging of the dynamics of plus end-binding proteins indicates that, as in other organisms, *C. elegans* axonal MTs are consistently arranged with plus ends away from the soma (‘plus end out’) but that dendritic MTs either are oriented with minus ends out or have mixed orientation ^37, 38^.

After axon injury, the stable axonal MTs become highly dynamic to allow axonal regrowth and establishment of a new growth cone ^6, 7^. In cultured *Aplysia californica* neurons, injury triggers rapid MT depolymerization followed by repolymerization with aberrant MT orientation ^39, 40^. Reversal of MT polarity after injury has been observed in Drosophila dendrites ^41, 42^. In addition, axotomy triggers an acute change of MT dynamics in Drosophila ^41, 43, 44^. In *C. elegans*, axotomy of the mechanosensory posterior lateral microtubule (PLM) neuron triggers an increase in growing MTs locally at the injury site, followed by persistent growth of MTs that leads to formation of functional growth cones ^37^. A mutation in *mec-7*/β-tubulin that hyperstabilizes MTs in touch neurons inhibits anterior lateral microtubule (ALM) axon regeneration, suggesting that precise regulation of MT dynamics is essential for axon regeneration ^45^. Regenerating axon tips in severed mouse neurons display an acute increase in MT dynamics, followed by a sustained increase over several days ^46^. Collectively, these findings suggest that axonal injury initiates an intricate series of changes in axonal MT organization.

## The DLK-1 MAPK cascade promotes axon regeneration, in part via microtubule dynamics

The DLK MAPK pathway was identified several years ago as essential for axon regeneration in *C. elegans* motor neurons and in mechanosensory neurons ^21, 22^. Mutants lacking DLK-1 [ *dlk-1(lf)*] display normal developmental axon growth but are unable to regenerate after injury, being blocked at the initial phase of growth cone reformation ( Figure 1B). Conversely, overexpression of *dlk-1* [ *dlk-1(gf)*] enhances axon regeneration ^21, 22^. A mammalian DLK-1 homolog MAP3K13/LZK can functionally substitute for *dlk-1* ^47^, suggesting a high degree of conservation of the DLK pathway in axon regeneration. Indeed, in mammals, DLK is also required for axon regeneration after axonal injury ^26– 29^.

DLK-1 activity is required cell-autonomously at the time of regrowth, and DLK-1 itself is likely activated by injury signals. An axotomy-triggered Ca ^2+^ transient has been implicated in DLK-1 activation ^47– 49^. In addition, the DLK pathway is sensitive to MT depolymerization. Mutations disrupting MTs trigger a DLK-dependent reduction of protein levels in touch neurons ^50^. In Drosophila, loss of Short stop (shot), a member of the spectraplakin family that crosslinks actin and MT ^51, 52^, activates the DLK signaling pathway to promote axon regeneration ^53^. Moreover, disruption of MTs by nocodazole in mammalian sensory neurons activates the DLK signaling pathway ^54^. As yet, it remains unclear how MT polymerization is sensed by DLK.

Activation of the DLK pathway leads to two major outputs in *C. elegans*: a transcriptional response involving the CEBP-1 bZip transcription factor and CEBP-1-independent effects on axonal MT dynamics. The *dlk-1(lf)* mutant fails to increase persistent MT growth after axotomy, whereas *dlk-1(gf)* shows increased number of growing axonal MTs, both before and after axotomy ^37^. Following laser injury, the DLK pathway promotes MT dynamics and growth, through downregulation of the kinesin-13 KLP-7 and upregulation of the cytosolic carboxypeptidase CCPP-6 ^37^. Thus, the DLK cascade is closely interconnected with MTs, both as a sensor of MT integrity and as a regulator of MT dynamics, making it well placed to mediate regenerative reorganization of the axonal MT cytoskeleton after injury.

## EFA-6, an inhibitor of axon regeneration acting via microtubule dynamics

The above studies of DLK-1 have helped spur efforts to identify additional factors that control MT dynamics during axon regeneration. Using a large-scale genetic screen, we identified the evolutionarily conserved protein EFA-6 (exchange factor for Arf-6) as a cell-intrinsic suppressor of axon regeneration ^18^. Loss-of-function mutations of *efa-6* [ *efa-6(lf)*] enhance axonal regeneration, whereas *efa-6* overexpression [ *efa-6(gf)*] blocks regeneration ( Figure 1B). Uniquely, *efa-6(lf)* partially bypasses the requirement for DLK-1 in axonal regeneration ^18^, suggesting that EFA-6 and DLK-1 have antagonistic effects on a common process. Multiple lines of evidence suggest that EFA-6 inhibits axon regeneration through modulation of MT dynamics ^18, 55^.

**Figure 2.**
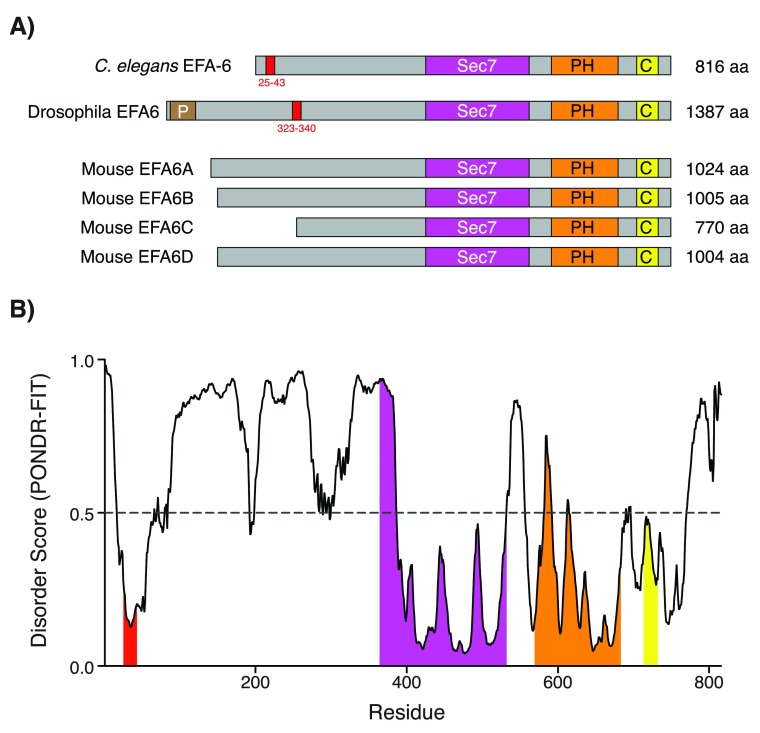
The intrinsically disordered N-terminal of EFA-6 contains a conserved 18-amino acid (18-aa) motif. ( **A**) EFA-6 protein domain organization in different organisms. Red boxes in the EFA-6 N-terminus highlight a conserved 18-aa motif, found in both *Caenorhabditis elegans* and Drosophila ^63^. ( **B**) Plot of intrinsic protein disorder score for *C. elegans* EFA-6. Different domains of EFA-6 are color-coded as in ( **A**). Note that EFA-6 N-terminus has an overall high disorder probability, apart from the 18-aa motif. Figure adapted from 55.

The EFA-6/EFA6 protein family is conserved from yeast to mammals. EFA-6 contains a Sec7 domain that confers guanine exchange activity (GEF) for Arf6 GTPases ^56^. Four EFA6 members (EFA6A–EFA6D) have been identified in mammals and three of them (except EFA6B) are expressed in neurons ^57, 58^. EFA6 localizes to the plasma membrane through its pleckstrin homology (PH) domain ^56^. Furthermore, EFA6 can interact with filamentous actin *in vitro* through its PH domain and plays important roles in regulation of cortical actin cytoskeleton in vertebrate cells ^56, 57, 59– 61^. In *C. elegans*, *efa-6* suppresses the embryonic lethality caused by mutations in dynein, a MT motor ^62^, suggesting a functional linkage between actin and MT cytoskeletons at the cell cortex. In the one-cell *C. elegans* embryo, EFA-6 localizes to the plasma membrane, with enrichment in the anterior cortex in late one-cell embryo, to limit MT growth throughout the cell cortex ^62, 63^. The plasma membrane localization of EFA-6 is dependent upon the presence of its PH domain, whereas the intrinsically disordered N-terminal domain confers the enrichment at the anterior cell cortex ^63^. The N-terminal region of EFA-6 contains a conserved 18-amino acid (18-aa) motif ( Figure 2), which is essential for the MT growth-inhibiting activity.

**Figure 3.**
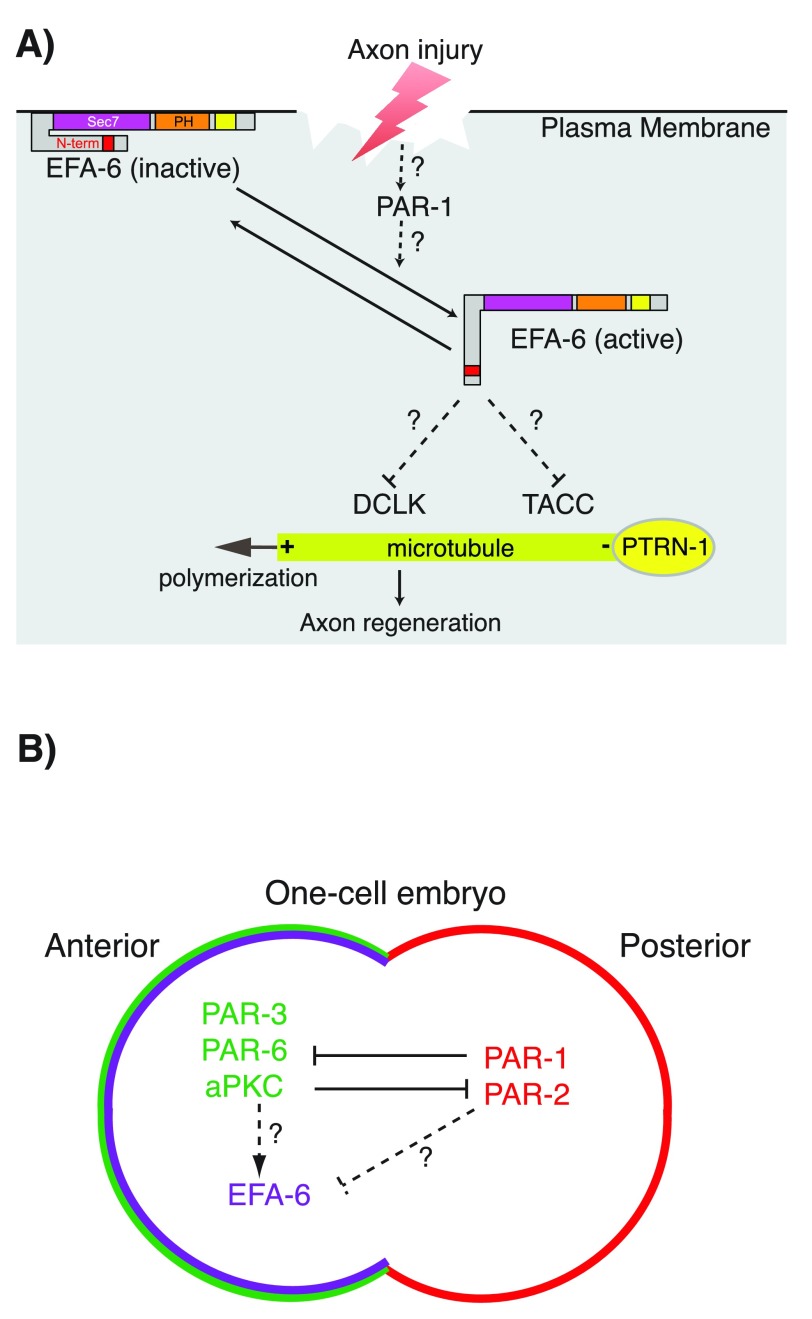
Functions of EFA-6 and PAR-1 in axon regeneration and polarity control in the early *Caenorhabditis elegans* embryo. ( **A**) Model for the regulation of EFA-6. In steady-state axons, EFA-6 localizes to the plasma membrane through its pleckstrin homology (PH) domain. Upon axon injury, EFA-6 and TAC-1/TACC (transforming acidic coiled-coil) relocalize close to the microtubule (MT) minus ends as defined by PTRN-1/Patronin puncta. The N-terminal intrinsically disordered region of EFA-6 is necessary and sufficient for relocalization and binding to TAC-1. ( **B**) In one-cell embryos, PAR-1 and PAR-2 localize to the posterior cortex. This localization restricts the PAR-3/PAR-6/aPKC polarity complex to the anterior cortex. EFA-6 is enriched at the anterior cortex, dependent on the intrinsically disordered N-terminal domain. Such polarity complexes could regulate EFA-6 localization.

In mature uninjured neurons, EFA-6 also localizes to the cell cortex via its C-terminal PH domain ^55^. Upon axotomy, EFA-6 rapidly (within minutes) relocalizes to puncta close to sites containing MT minus ends, as marked by the minus end-binding protein PTRN-1/Patronin ^55, 64^. Relocalization of EFA-6 is dependent on the intrinsically disordered N-terminal domain and plays important roles in inhibition of axon regeneration ( Figure 3A). In addition, the N-terminal domain of EFA-6 binds to the MT-associated proteins TAC-1/TACC (transforming acidic coiled-coil) and ZYG-8/DCLK (double-cortin-like kinase). Both TAC-1 and ZYG-8 are required for regenerative growth cone formation after axonal injury ^55^. Although the roles of mammalian EFA6 family members in axon regeneration remain to be examined, *Xenopus* TACC3 promotes axon outgrowth in embryonic cultured neural crest cells ^65^, and DCLK is required in mammalian axon regrowth ^66^, suggesting potential functional conservation from *C. elegans* to mammals.

## EFA-6, cell polarity proteins, and microtubule dynamics

Current evidence suggests that EFA-6 has bifunctional roles, as an MT destabilizing factor at the cell cortex in the steady state and relocalizing to the vicinity of MT minus ends after injury ^55^. The molecular mechanisms involved in the transitions between these putative EFA-6 activity/localization states remain unknown. However, the intrinsically disordered N-terminal domain appears to be key to understanding the functions of EFA-6 with respect to MT dynamics. All EFA6 family members contain large N-terminal domains that are predicted to be intrinsically disordered ( Figure 2A). Within the N-terminal domain, the sole region of primary sequence conservation is the 18-aa motif conserved in *C. elegans* and other invertebrate EFA6 family members and partly recognizable in some vertebrate EFA6 members. This motif contains potential phosphorylation sites, mutation of which abolishes the relocalization and regeneration-inhibiting activities of EFA-6 ^55^. However, the identity of upstream kinases or phosphatases remains unknown; DLK-1 does not appear to be required for injury-triggered relocalization of EFA-6.

As the phosphorylation status of EFA-6 is tightly correlated to its MT dynamics-regulating activity ^55^, the kinase or kinases responsible for EFA-6 phosphorylation may also have MT dynamics-regulating activity, directly or indirectly. Furthermore, like EFA-6, such kinases may also regulate MT dynamics in early embryo. Many candidates, including PAR-1/MARK (MT affinity-regulating kinase) and polo-like kinase, have been implicated in MT dynamics in embryos and neurons ^67, 68^, although none of these kinases has yet been associated with EFA-6 phosphorylation. Here, we focus on PAR-1/MARK and summarize the known functions of PAR-1/MARK in neurons and embryos that may be relevant to axon regeneration.

PAR-1/MARK is one of the PAR (partitioning defective) proteins, first identified in *C. elegans* for their roles in polarization of the early embryo ^69^. PAR-1 encodes a serine/threonine kinase related to the MARKs ^70^. Misregulation of PAR-1 and its phosphorylation targets has long been implicated in neuronal diseases such as Alzheimer’s disease and autism ^71, 72^. In mammalian neurons, MARK phosphorylates the MT-associated proteins (MAPs) tau, MAP2, and MAP4, causing these MAPs to dissociate from MTs and thereby destabilizing the MT network and increasing MT dynamics ^70^. Furthermore, expression of MARK in cultured neurons promotes neurite outgrowth ^73^. Neurite outgrowth involves a pioneer population of dynamic MTs that invades growth cones, followed by MT stabilization in axon extension ^74^. These studies suggest that PAR-1/MARK plays key roles in MT plasticity during neurite outgrowth; less is known of its roles in axon regeneration.

PAR-1/MARK can regulate MT dynamics in many cell types ^75– 79^. In *C. elegans* one-cell embryos, PAR-1, together with its partner PAR-2, accumulates at the posterior cortex ^80^. This localization prevents the anterior polarity complex, PAR-3/PAR-6/aPKC, from concentrating at the posterior cortex ^81, 82^ ( Figure 3B). Intriguingly, MTs are more dynamic at the posterior end of an embryo, dependent on the asymmetric distribution of PAR proteins, including PAR-1 ^75^. It is possible that PAR-1 regulates localization of EFA-6, causing an enrichment of EFA-6 at the anterior cortex ( Figure 3B). Interactions between PAR network proteins and EFA-6 in the early embryo have not yet been tested but could affect regulation of cortical MT dynamics.

## Concluding remarks

Recent studies have highlighted the importance of MT dynamics in regulation of axon regeneration. Several players, including DLK-1 and EFA-6, have been identified to regulate MT dynamics upon axon injury. However, many questions remain unexamined (see “Outstanding issues”). Importantly, pharmacological stabilization of MTs enhances axon regeneration both *in vitro* and *in vivo*, highlighting the therapeutic potential of MT dynamics regulation in axon regeneration. We anticipate that future studies should elucidate these mechanisms, which are potentially relevant to therapeutic interventions aimed at promoting regenerative axon growth.

## Outstanding issues

### 1. What triggers EFA-6 relocalization upon axon injury?

EFA-6 rapidly relocalizes close to the MT minus ends to inhibit MT dynamics upon axon injury. This relocalization may be controlled by phosphorylation of the intrinsically disordered N-terminus of EFA-6. However, the signals (kinases/phosphatases) that might trigger EFA-6 relocalization or function remain to be discovered. Identification of these signals will bring crucial insights into how the activity of EFA-6 is regulated, possibly allowing precise manipulation of EFA-6 activity in a regrowing axon.

### 2. What are the cellular targets of DLK and EFA-6?

Upon axotomy, DLK-1 promotes MT dynamics and growth, whereas EFA-6 relocalizes close to the MT minus ends to inhibit MT dynamics. Both proteins seem to affect axon regeneration by regulating MT dynamics. Intriguingly, *efa-6(lf)* can partially bypass the requirement of DLK-1 in axon regeneration. However, how EFA-6 interacts with the DLK-1 pathway remains unclear. It is of great interest to investigate these issues further to provide a better understanding of how MT dynamics control axon regeneration.

### 3. Is EFA-6 a conserved inhibitor of axon regeneration?

Identification of EFA-6 as a cell-intrinsic inhibitor of axon regeneration in *C. elegans* raises the question of whether any of the four mammalian EFA6 family members are involved in mammalian axon regeneration or axonal MT dynamics or both. Like *C. elegans* EFA-6, mammalian EFA6 family members all contain large N-terminal domains that are predicted to be intrinsically disordered. EFA6A, EFA6C, and EFA6D are expressed in the nervous system, but their roles in axon regeneration have yet to be assessed. Given evidence that partial stabilization of MT dynamics can improve axon regeneration in vertebrates, manipulation of specific MT destabilizing factors such as EFA-6 might allow a more targeted approach to enhancing regrowth in a therapeutic context.
